# Leptin Signaling in the Carotid Body Regulates a Hypoxic Ventilatory Response Through Altering TASK Channel Expression

**DOI:** 10.3389/fphys.2018.00249

**Published:** 2018-03-27

**Authors:** Fang Yuan, Hanqiao Wang, Jiaqi Feng, Ziqian Wei, Hongxiao Yu, Xiangjian Zhang, Yi Zhang, Sheng Wang

**Affiliations:** ^1^Department of Physiology, Hebei Medical University, Shijiazhuang, China; ^2^Hebei Key Laboratory of Vascular Homeostasis and Hebei Collaborative Innovation Center for Cardio-Cerebrovascular Disease, Shijiazhuang, China; ^3^Department of Sleep, Third Hospital of Hebei Medical University, Shijiazhuang, China; ^4^Department of Neurology, Second Hospital of Hebei Medical University, Shijiazhuang, China

**Keywords:** leptin, hypoventilation, hypoxic ventilatory response, carotid body, TASK channels, STAT3

## Abstract

Leptin is an adipose-derived hormone that plays an important role in the regulation of breathing. It has been demonstrated that obesity-related hypoventilation or apnea is closely associated with leptin signaling pathways. Perturbations of leptin signaling probably contribute to the reduced sensitivity of respiratory chemoreceptors to hypoxia/hypercapnia. However, the underlying mechanism remains incompletely understood. The present study is to test the hypothesis that leptin signaling contributes to modulating a hypoxic ventilatory response. The respiratory function was assessed in conscious obese Zucker rats or lean littermates treated with an injection of leptin. During exposure to hypoxia, the change in minute ventilation was lower in obese Zucker rats than chow-fed lean littermates or high fat diet-fed littermates. Such a change was abolished in all groups after carotid body denervation. In addition, the expression of phosphorylated signal transducers and activators of transcription 3 (pSTAT3), as well as putative O_2_-sensitive K^+^ channels including TASK-1, TASK-3 and TASK-2 in the carotid body, was significantly reduced in obese Zucker rats compared with the other two phenotype littermates. Chronic administration of leptin in chow-fed lean Zucker rats failed to alter basal ventilation but vigorously increased tidal volume, respiratory frequency, and therefore minute volume during exposure to hypoxia. Likewise, carotid body denervation abolished such an effect. In addition, systemic leptin elicited enhanced expression of pSTAT3 and TASK channels. In conclusion, these data demonstrate that leptin signaling facilitates hypoxic ventilatory responses probably through upregulation of pSTAT3 and TASK channels in the carotid body. These findings may help to better understand the pathogenic mechanism of obesity-related hypoventilation or apnea.

## Introduction

Leptin, a peptide hormone secreted mainly by adipocytes, regulates multiple physiological functions including metabolism, cardiovascular activity, and breathing (Grill et al., 2002; Bassi et al., 2015). Leptin's role in controlling breathing has been implicated in recent studies, with its participation in sleep-related breathing disorders including obesity hypoventilation syndrome (OHS) and obstructive sleep apnea (Malhotra and White, 2002). In animal models, leptin deficient mice exhibit impaired ventilatory responses to CO_2_ which can be rescued by leptin replacement therapy, in favor of facilitation of breathing by leptin (O'Donnell et al., 2000). Leptin receptors (ob-Rs) are composed of six isoforms termed from ob-Ra to -Rf, with the long form of ob-Rb mediating the majority of leptin's intracellular signal transduction (Tartaglia, 1997). Although the role of leptin signaling pathways in mediating various physiological actions has been investigated intensively, the molecular mechanism underlying its action on breathing remains incompletely understood.

The peripheral respiratory chemoreflex serves as a homeostatic regulatory mechanism by which enough oxygen must be supplied to the organism when challenged by hypoxia through altering respiratory amplitude and frequency. The carotid body (CB) chemoreceptors, located near the fork of the carotid artery, are activated shortly after exposure to hypoxia and then send information to the nucleus tractus solitarius and high integrative centers, with the outcome of adaptive ventilatory responses (Gonzalez-Martin et al., 2011; Ciriello and Caverson, 2014). Accumulated evidence indicates the presence of ob-Rb in the CB cells (Porzionato et al., 2011; Messenger et al., 2013), and that leptin signaling contributes to CB-mediated ventilatory responses (Olea et al., 2015; Ribeiro et al., 2017). However, it remains controversial whether the CB mediates the acute effect of leptin on hypoxic ventilatory response (HVR) because leptin's role may involve the change in gene expression and protein synthesis, requiring hours even days for full effects (Hall et al., 2010). We thereby predicted that the stimulatory effect of leptin on HVR may require chronic activation of CBs, but such a confirmation is yet to be put forward.

In the CBs, leptin signaling pathways involve the downstream signaling proteins of ob-R signal transducers and activators of transcription 3 (STAT3), suppressor of cytokine signaling 3 (SOCS3), and extracellular-signal-regulated kinase 1/2 (ERK1/2) (Messenger et al., 2013; Moreau et al., 2015), reminiscent of modulatory effects of these molecules on carotid chemoreceptor sensitivity. Emerging evidence has shown that the chemosensitivity of glomus cells in CBs requires two-pore K^+^ channels including TWIK-related acid-sensitive K (TASK)-1 channels and acid-sensitive ion channels (Trapp et al., 2008; Tan et al., 2010). However, very little is known concerning whether the activation of the ob-R and downstream signaling molecules modulates the sensitization of CB chemoreceptors via affecting these ion channels.

We sought to address herein whether the leptin signaling pathway in the CB contributes to regulating HVR and the possible mechanism involved. We utilized whole body plethysmography (WBP) to assess HVR in obese Zucker rats (ob-R deficiency) or in lean littermate controls treated with injections of leptin. The main findings suggest that chronic application of leptin contributes to facilitation of HVRs probably through upregulation of phosphorylated STAT3 (pSTAT3) and TASK channel expression.

## Materials and methods

### Animals

The experiments were carried out in 12~20-week-old male obese Zucker rats (OZR) and lean littermates (LZR) obtained from the Charles River Laboratories (USA). Animals, synchronized for a 12:12 h light-dark cycle (lights on at 8 am, lights off at 8 pm), were housed individually and allowed to move freely in standard plastic cages in a climate-controlled room (22 ± 1°C). Food and water were provided *ad libitum* for LZRs and OZRs. In some cases, a group of LZRs were placed on a high-fat diet (HFD, 45% kcal/g fat, Research Diets D12451) for 8 weeks and used as a simple obesity control (LHZR). The LHZR and OZR groups were weight-matched to determine the effect of simple obesity-induced mechanical resistance on ventilation. Body weight was measured once a week (*n* = 20 for each phenotype). All experiments were performed in accordance to ethical guidelines of the Animal Protection Association and were approved by Animal Care and Ethical Committee of Hebei Medical University. When the animal experiments were completed, an overdose of intraperitoneal injection of sodium pentobarbital (> 200 mg/kg) was carried out for euthanasia.

### Breathing measurement

Breathing was studied by WBP in conscious, freely moving rats (EMKA Technologies, France) as described previously (Kumar et al., 2015; Fu et al., 2017). In brief, rats were placed in the WBP chamber on the day before the testing protocol (2 h acclimation period). For acute hypoxia, rats were exposed to 10% O_2_ (balance N_2_) for up to ~7 min by a gas mixture devices (1,500 ml/min, GSM-3, CWE, USA). Ventilatory flow signals were recorded, amplified, digitized and analyzed using IOX 2.7 (EMKA Technologies) to determine breathing parameters over sequential 20 s epochs (~50 breaths) during periods of behavioral quiescence and regular breathing. Minute volume (V_E_; ml/min/g) was calculated as the product of the respiratory frequency (f_R_, breaths/min) and tidal volume (V_T_; ml/kg), normalized to rat body weight (g). To further confirm the CB-mediated effect of leptin, breathing parameters were also measured in rats with carotid body denervation (CBD). The carotid sinus nerves were sectioned as depicted before (Kumar et al., 2015). Shortly, anesthesia was induced with 4% halothane in 100% O_2_ and maintained by reducing the inspired halothane concentration to 1.5~1.8%. The depth of anesthesia was assessed by an absence of the corneal and hindpaw withdrawal reflex. Body temperature of all mice was maintained at 37°C using a temperature-controlled heating pad. To prevent any functional regeneration of chemosensory fibers, the carotid sinus nerves were removed completely from the cranial pole of the CB until reaching the branch to the glossopharyngeal nerve. The wound was carefully sutured and disinfected with 10% of polividone iodine. Conscious chemodenervated rats were exposed to ventilatory challenge 5–7 days after recovery. No significant weight loss was observed. All the three groups of rats (LZR, LHZR and OZR) were submitted to surgery.

To examine whether hypoventilation resulted in retention of CO_2_ in obese rats, arterial blood gas was measured using an OPTI-CCA blood gas analyzer (OPTI Medical Systems, USA) at a steady state in halothane-anesthetized, paralyzed rats. General anesthesia was induced with 4% halothane in room air as depicted above. Arterial blood (200 μl per sample) was drawn from the femoral artery in the three animal groups. Arterial blood measurements of interest included partial pressure of arterial O_2_ (P_a_O_2_), partial pressure of CO_2_ (P_a_CO_2_) and pH.

### Plasma leptin levels and hypodermic leptin injections

Measurements of plasma levels of leptin were performed at room air (21% O_2_) in anesthetized rats. After general anesthesia as described above, whole blood samples were taken through a cardiac puncture. Blood samples were drawn into collection tubes containing the anticoagulant EDTA (Sigma-Aldrich, USA) and kept on ice. After centrifugation, the plasma was stored at−80°C for leptin analysis by ELISA kit (#ab100773, Abcam, USA), an *in vitro* enzyme-linked immunosorbent assay for the quantitative measurement as previously described (Panetta et al., 2017). The assay was read using a power wave XS2 plate reader (Biotek Instruments, USA).

To confirm whether the chronic activation of leptin signaling pathways played a part in the HVR, subcutaneous injections of leptin (60 μg/kg) or equal volume of vehicle (saline) were carried out once daily for 7 days in CBI LZRs (*n* = 8 for each group), and breathing parameters were measured after 7 day injections during exposure to room air or hypoxia. To further confirm the CB's role, subcutaneous injections of leptin or saline were performed 7 days after the carotid sinus nerves were sectioned in each group (*n* = 8 for both).

### Carotid body protein extracts and western blot analysis

Rats were deeply anesthetized by isoflurane (2–3%) inhalation and then decapitated. The carotid bifurcation was exposed and both CBs were removed and cleaned. The CB (*n* = 8) were pooled from 4 rats in each group and homogenized in 100 μl of RIPA buffer solution (150 mM NaCl, 1 mM EDTA, 1% Triton-X 100, 50 mM Tris–HCl at pH of 7.5) with a protease inhibitor cocktail (Roche Diagnostics, Canada). The homogenate was centrifuged at 4°C for 20 min at 13200 rpm. The resultant supernatant was retained as the protein preparation. Equal concentrations of extracted proteins normalized by colorimetric BCA Protein Assay (Pierce Corp., USA). After denaturation, the protein (~30 μg) in each lane was fractionated in 10% polyacrylamide gel and then transferred onto apolyvinylidene fluoride membrane. The membranes were blocked with bovine serum albumin and incubated at 4°C overnight with primary antibodies anti-ob-R (1:2000, #ab5593, Abcam, USA), anti-TASK-1 (1:200, #APC024, Alomone labs, Israel), anti-TASK-2 (1:200, #APC037, Alomone labs, Israel), anti-TASK-3 (1:200, #APC044, Alomone labs, Israel), anti-pSTAT3 (1:2000, #9145, Cell Signaling Technology, USA), anti-STAT3 (1:1000, #9139, Cell Signaling Technology, USA), and anti-β-actin (1:3,000, #T0022, Affinity Biosciences, USA). The membranes were then incubated with corresponding secondary antibodies for 1 h at room temperature. The reaction was visualized using the enhanced chemiluminescence (ECL) method, and the bands were analyzed by ImageJ software (NIH, USA). The protein contents were normalized to β-actin. See Supplementary Material for original gel images.

### Data acquisition and processing

Data are expressed as mean ± SEM. Unless indicated otherwise, two-tailed unpaired *t*-test, one-way ANOVA with Dunnett's or Tukey's *post-hoc* test and two-way ANOVA with Bonferroni *post-hoc* test were used to compare significant difference between different groups. Differences within or between groups with *P*-values of < 0.05 were considered significant.

## Results

### Reduced basal ventilation in OZRs

Adult OZRs exhibit many abnormal physiological attributes due to the deficiency of the ob-R, representing a good animal model to study the obesity-related hypoventilation as observed in humans. We thus addressed leptin's role using this phenotype and the littermate control rat. First, we measured the body weight of three groups of rats (*n* = 20 for each group). The averaged body weight was larger in LHZRs (492 ± 30 g) and OZRs (512 ± 49 g) than LZRs (382 ± 14 g, *P* < 0.01 vs. LHZR or OZR). In addition, weight gain was accompanied by higher levels of blood plasma leptin level in OZRs (15.28 ± 0.55 μg/L) in relative to LHZRs (7.45 ± 0.50 μg/L, *P* < 0.01 vs. OZR) or LZRs (7.34 ± 0.38 μg/L, *P* < 0.01 vs. OZR; *P* > 0.05, vs. LHZR).

**Figure 1:**
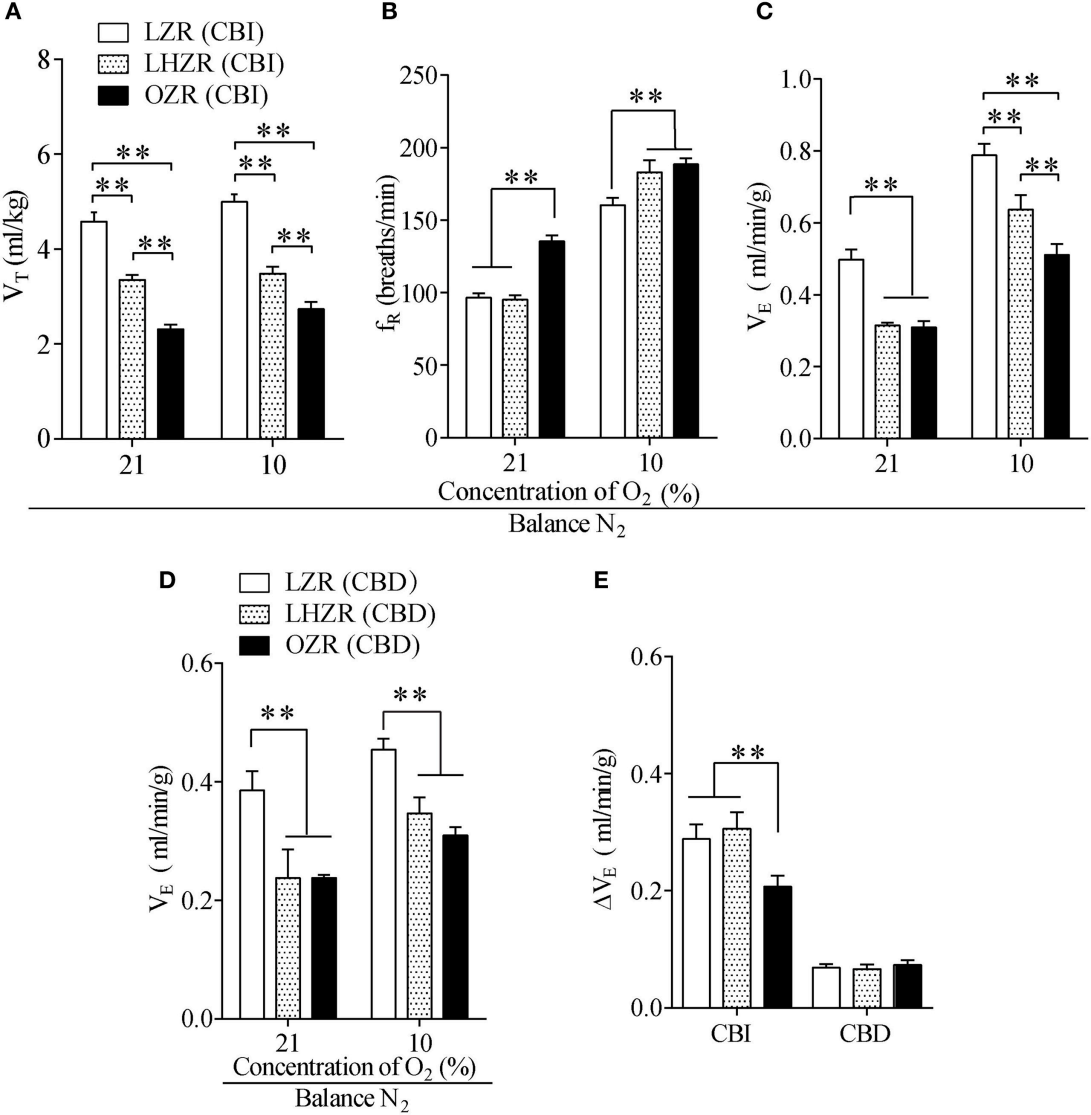
The OZR exhibits impaired HVR. **(A–C)** Effect of hypoxia on V_T_, f_R_, and V_E_ in the three groups of rats. **(D)** The stimulatory effect of hypoxia on V_E_ in three groups of CBD rats. **(E)** Changes in V_E_ during exposure to 10% O_2_ between CBI and CBD rats. *n* = 20 for each group, ^**^P < 0.01 as drawn by two-way ANOVA with Bonferroni *post-hoc* test. V_T_, tidal volume; f_R_, respiratory frequency; V_E_, minute ventilation; CBI, carotid body innervation; CBD, carotid body denervation.

Baseline breathing parameters were measured in the three groups of animals while breathing room air (21% O_2_). Compared with the LZRs, V_E_ and V_T_ were considerably lower in OZRs and LHZRs (*P* < 0.01 for both, vs. LZRs, Figures 1A,C), whereas the OZR has a faster f_R_ than the other two groups (*P* < 0.01 for both). Of interest, although no remarkable difference in basal V_E_ was observed between OZRs and LHZRs, V_T_ and f_R_ were comparable (*P* < 0.01 for both, Figure 1B), indicative of a different breathing pattern. To evaluate whether hypoventilation resulted in hypercapnia or respiratory acidosis in obese animals, the arterial blood gas was measured at a steady-state. Apparently, hypercapnia, acidosis and normal P_a_O_2_ were observed in LHZRs and OZRs (Table 1). Therefore, both OZRs and LHZRs exhibit basal hypoventilation, overweight and hypercapnia, with the exception of hyperleptinemia in OZRs but not LHZRs.

**Table 1:** Blood gas analysis during exposure to room air.

**Group**	**P_a_CO_2_(mmHg)**	**P_a_O_2_(mmHg)**	**pH**
LZR	37.9 ± 1.8	102.5 ± 5.9	7.32 ± 0.02
LHZR	51.6 ± 1.0^**^	93.8 ± 2.7	7.24 ± 0.01^*^
OZR	50.9 ± 0.6^**^	87.6 ± 2.8^*^	7.22 ± 0.01^*^

*LZR, lean Zucker rats; LHZR, HFD-fed LZR; OZR, obese Zucker rats; n = 12 for each group*;

* *P < 0.05*,

** *P < 0.01 by one-way ANOVA with Tukey's post-hoc test, vs. LZRs*.

### Impairment of HVR in OZRs

To address whether the ob-R deficiency yielded a diminished HVR, hypoxia was achieved while inhaling 10% O_2_ to activate peripheral respiratory chemoreflex. When acutely challenged with hypoxia, all three phenotypes displayed robust increases in f_R_ and V_E_ except for V_T_, with the smallest change in the HVR in OZRs (Figures 1A–C). In addition, the hypoxia-stimulated increment of V_E_ was far less in OZRs compared to the other two groups (*P* < 0.01, Figure 1E). Interestingly, LZRs and LHZRs displayed a similar change in V_E_ during hypoxia (*P* > 0.05, Figure 1E). In spite of similar degree of body weight, OZRs exhibited far more severe hypoventilation in response to hypoxia compared to LHZRs (*P* < 0.01, Figures 1A–C). However, exposure to hypoxia caused no significant difference in increments of V_E_ in all three groups of rats after sectioning carotid sinus nerves (*P* > 0.05, Figures 1D,E), in favor of the involvement of CBs in such an effect. Collectively, the ob-R deficiency (OZR), instead of simple obesity (LHZR), plays a predominant role in the impaired HVR.

### Downregulation of pSTAT3 and TASK channels in OZR CBs

**Figure 2:**
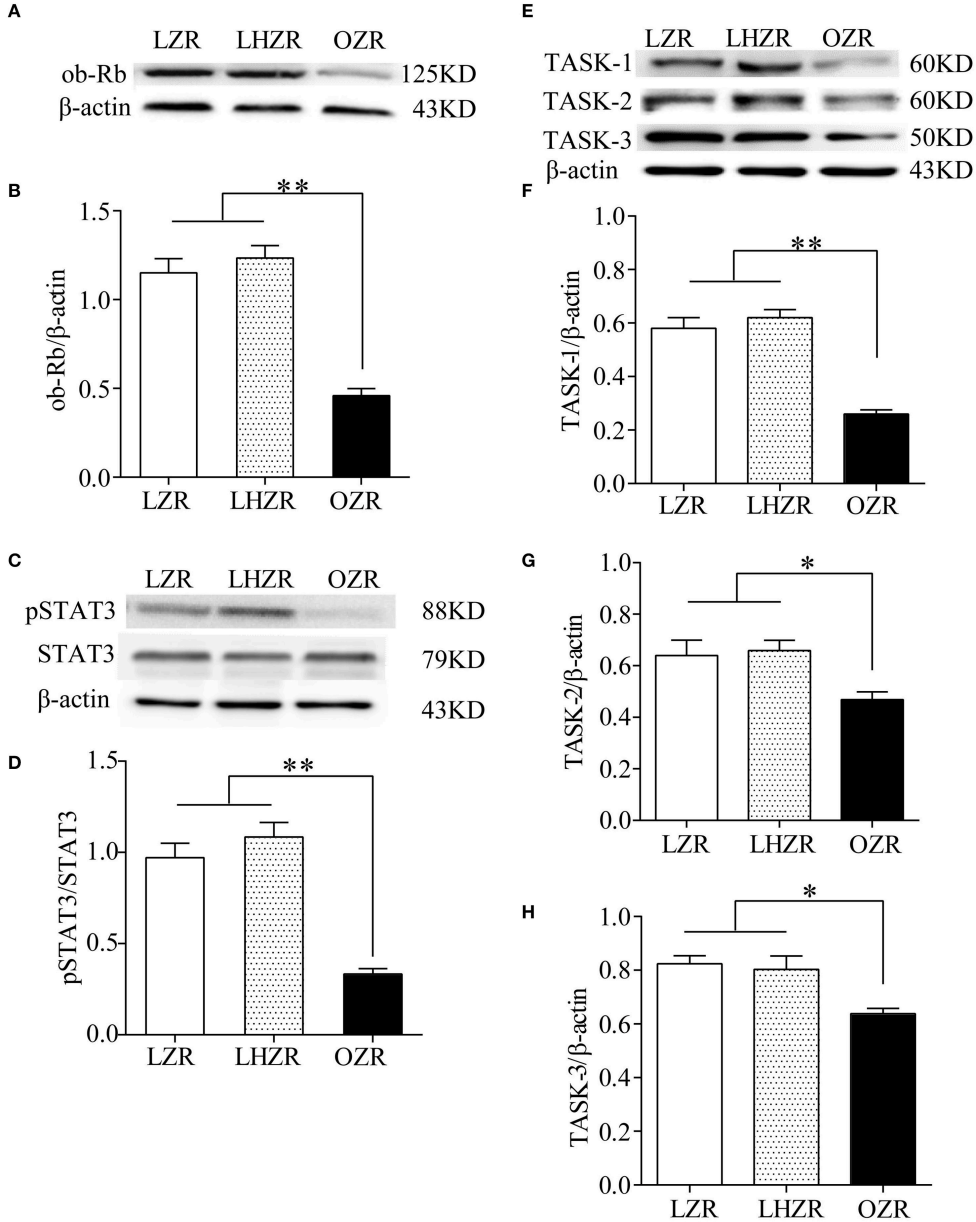
Downregulation of pSTAT3/STAT3 and TASK-1,-2,-3 channels in CBs. Gel images and bar charts showing quantitative analysis of expression of ob-Rb **(A,B)**, pSTAT3/STAT3 **(C,D)**, and TASK-1,-2,-3 **(E–H)** in the CB (*n* = 8) from each group (*n* = 4). ^*^P < 0.05, ^**^P < 0.01 by one-way ANOVA with Dunnett's *post-hoc* test. ob-Rb, leptin receptor type b; STAT3, signal transducers and activators of transcription 3; pSTAT3, phosphorylated STAT3.

The pSTAT3/STAT3 signaling has been implicated in mediating major effects of the ob-R. To determine the expression level of ob-R and STAT3, the quantitative analysis was made using Western blot in the three groups (*n* = 4 for each group). Compared with LZRs and LHZRs, pSTAT3/STAT3 (Figures 2C,D) were remarkably downregulated in the CBs of OZRs (*P* < 0.01), reliably correlating pSTAT3/STAT3 expression with the ob-R deficiency (Figures 2A,B). Several lines of evidence demonstrated that the chemosensitivity is associated with TASK-1 and TASK-3 in CB glomus cells and TASK-2 in retrotrapezoid nucleus neurons (Trapp et al., 2008; Gestreau et al., 2010; Wang et al., 2013). The reduced sensitivity of CBs to hypoxia in OZRs was probably associated with these ion channels. Evidently, the expression level of TASK-1, TASK-2, TASK-3 in OZR CBs was lower in relative to the other two groups (*P* < 0.05~0.01, Figures 2E–H). However, no statistical significance in these channel expression was found between LZRs and LHZRs (*P* > 0.05 for all, Figures 2E–H). Hence, the ob-R deficiency contributes to reduced expression of pSTAT3 and TASK channels.

### Facilitation of HVR by chronic application of leptin

**Figure 3:**
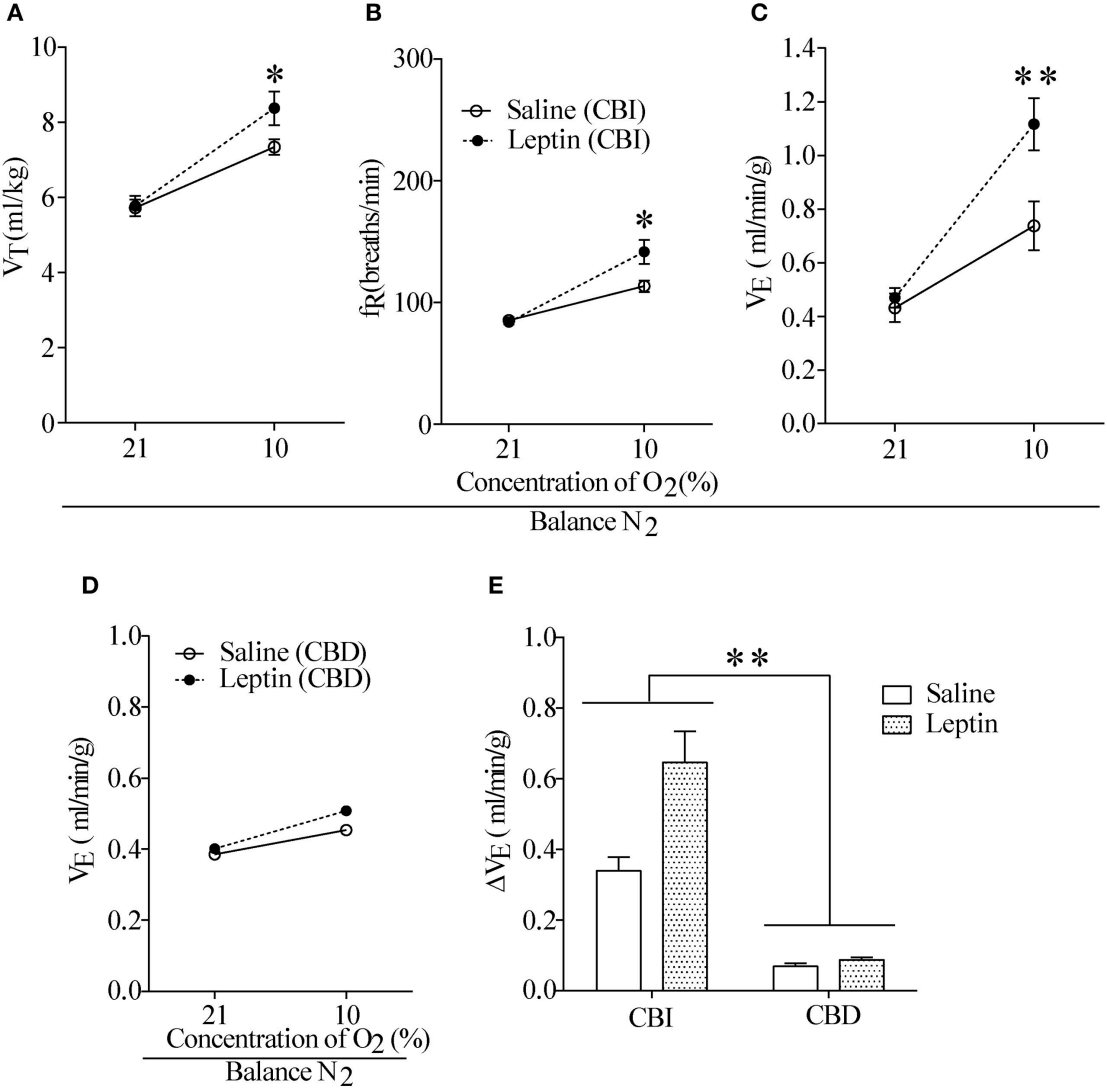
Effect of hypodermic injection of Leptin on breathing parameters. **(A–C)** Injection of leptin raised V_T_, f_R_, and V_E_ measured on 7th day in the LZR with CBI. **(D)** The stimulatory effect of hypoxia was reduced in two groups of rats with CBD. **(E)** Changes in V_E_ during exposure to 10% O_2_ in CBI and CBD groups. *n* = 8 for each group, ^*^P < 0.05, ^**^P < 0.01 by two-way ANOVA with Bonferroni *post-hoc* test, Leptin vs. Saline group.

To examine the effect of activation of ob-Rs on the HVR, subcutaneous injections of leptin (60 μg/kg) or equal volume of normal saline were carried out once daily for 7 days in LZRs (*n* = 8 for each group), and breathing parameters were measured at different time points separated by 7 days. As shown in Table 2, compared with the vehicle control (8.2 ± 0.4 μg/L), the plasma levels of leptin was raised to 13.1 ± 0.6 μg/L (*P* < 0.01) over 7-day treatment and restored to control level after 1 week. Chronic administration of leptin for 7 days produced no significant change in body weight (*P* > 0.05, data not shown) and basal breathing parameters (V_T_, f_R_ and V_E_) in relative to the vehicle control (Figures 3A–C). Neither P_a_CO_2_ nor blood pH changed significantly in leptin-injected rats (data not shown). During exposure to 10% O_2_, V_T_, f_R_, and V_E_ were all increased in either leptin- or saline-injected LZRs (*P* < 0.05~0.01) but the change in V_E_ was greater in leptin-injected rats in relative to the vehicle controls (Figure 3E). The stimulatory effect of leptin on HVR persisted for at least 2 weeks (Table 3). After bilaterally sectioning carotid sinus nerves, leptin-induced increase in V_E_ was abolished (Figures 3D,E). Collectively, chronic administration of leptin potentiated the HVR.

**Table 2:** Blood plasma levels of leptin in response to chronic application of saline and leptin.

**Day**	**Saline group (Leptin, μg/L)**	**Leptin group (Leptin, μg/L)**
0 day	7.8 ± 0.5	7.6 ± 0.5
7th day	8.2 ± 0.4	13.1 ± 0.6^**^
14th day	7.6 ± 0.5	9.3 ± 0.6
21st day	8.0 ± 0.6	8.6 ± 1.0

*n = 8 for each group*;

** *P < 0.01 by two-tailed unpaired t-test, vs. saline*.

**Table 3:** V_E_ measured during exposure to room air and hypoxia.

**Day**	**O_2_ level**	**Saline group (V_E_ ml/min/g)**	**Leptin group (V_E_ ml/min/g)**
0 day	Air	469 ± 41	451 ± 21
	10%O_2_	810 ± 24	819 ± 26
7th day	Air	479 ± 26	470 ± 36
	10%O_2_	819 ± 44	1117 ± 97^**^
14th day	Air	463 ± 17	481 ± 32
	10%O_2_	819 ± 32	1036 ± 88^**^
21st day	Air	468 ± 36	438 ± 24
	10%O_2_	771 ± 75	914 ± 48

*V_E_, minute ventilation, n = 8 for each group*,

** *P < 0.01 by two-way ANOVA with Bonferroni post-hoc test, vs. Saline*.

### Effect of leptin on expression of pSTAT3 and TASK channels

**Figure 4:**
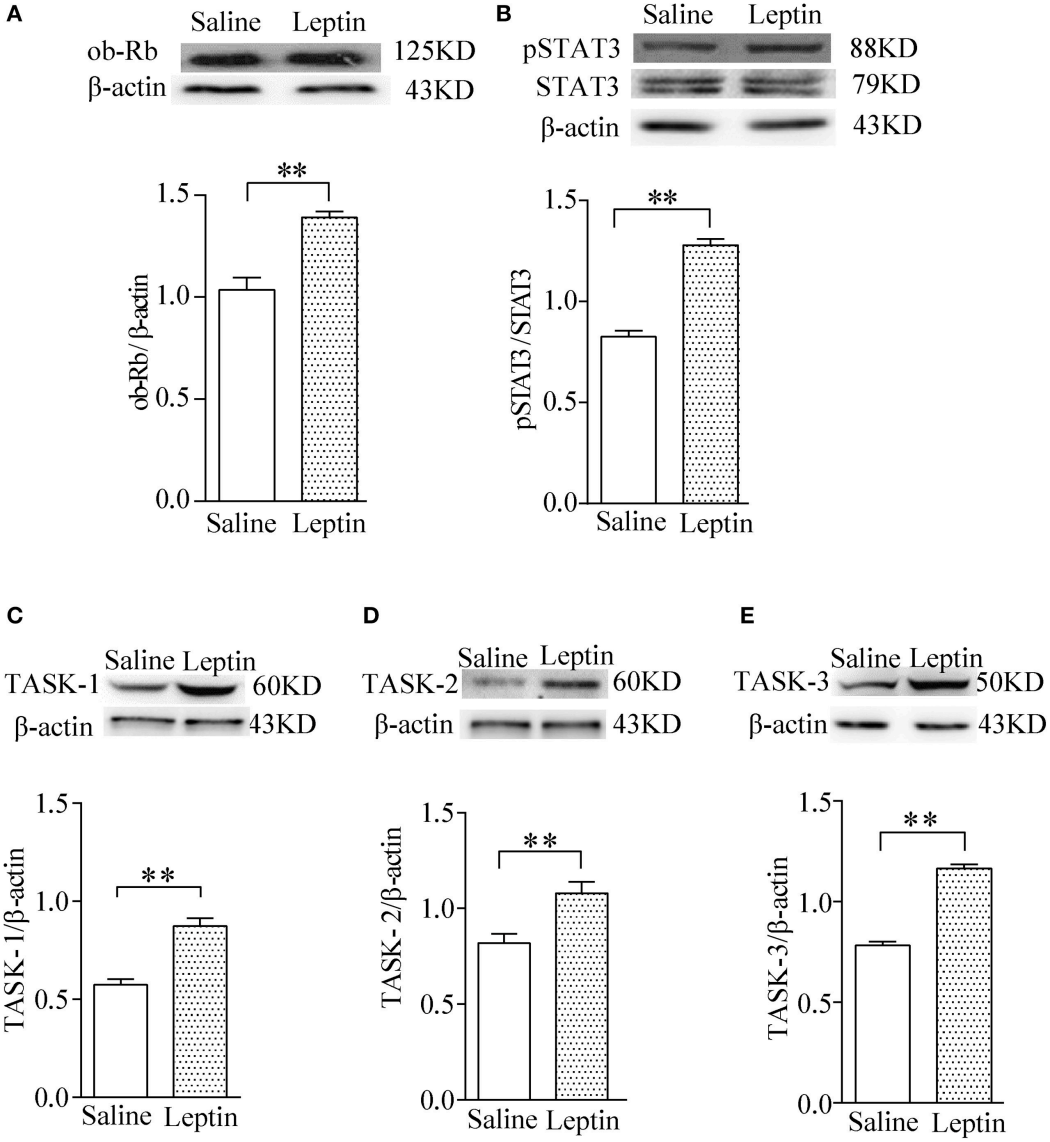
Stimulatory effect of leptin on pSTAT3/STAT3 and TASK-1,-2,-3 channel protein expression in CBs. Gel images and bar charts showing quantitative analysis of leptin-stimulated expression of ob-Rb **(A)** pSTAT3/STAT3 **(B)** and TASK-1,-2,-3 **(C–E)** in the CB (*n* = 8) of each group (*n* = 4). ^**^P < 0.01 by two-tailed unpaired *t*-test.

To investigate the possible mechanism underlying exogenous application of leptin action on the HVR, we tested the expression of ob-R and the downstream pSTAT3 and TASK-1, TASK-2, TASK-3 channels in CBs after injection of leptin for 7 days. The findings indicated that leptin administration caused greater upregulation of ob-R and of pSTAT3 (*P* < 0.01, *n* = 4 for each group, Figures 4A,B). Furthermore, leptin also enhanced expression of TASK-1, TASK-2, TASK-3 (*P* < 0.01, *n* = 4 for each group, Figures 4C–E). The results suggest that the stimulatory effect of leptin on HVR are closely associated with enhanced expression of pSTAT3 and TASK channels, which may contribute to the regulation of CB's chemosensitivity.

## Discussion

We demonstrate herein that the ob-R deficiency, rather than simple obesity, not only reduces baseline ventilation but also inhibits the HVR, with decreased pSTAT3 expression in CBs. Chronic administration of leptin has no marked effects on basal ventilation but considerably enhances the HVR, accompanied by increased expression of pSTAT3. Additionally, either ob-R deficiency or leptin administration is reliably associated with changes in expression of TASK-1, TASK-2, and TASK-3 channels. These findings suggest that leptin signaling in the CB contributes to potentiation of HVR probably through enhancement of pSTAT3 and TASK channels expressions.

The obese Zucker rat represents a good model of ob-R deficiency and manifests relatively early onset obesity (Bray and York, 1971). One line of early evidence shows that respiratory system compliance was significantly lower in the OZR compared with the lean phenotype, and that resting ventilatory parameters (uncorrected for body weight) were similar between obese and lean animals, with similar ventilatory response to hypoxia between two phenotypes (Farkas and Schlenker, 1994). In the present study, we compared the difference between obese and lean phenotypes using the previously described method (Kumar et al., 2015; Fu et al., 2017) to normalize breathing parameters to body weight. Interestingly, with this analysis method, the OZRs exhibited fast f_R_, reduced V_T_ and thus lower V_E_ during exposure to room air. This outcome interprets occurrence of hypercapnia and respiratory acidosis observed in OZRs. Based on these attributes, the OZR resembles an animal model of leptin resistance. The LHZR, a simple obesity control carrying genotype of the LZR, displayed hypercapnia, respiratory acidosis, relatively normal serum leptin, basal hypoventilation and moderate response to hypoxia, probably representing a model of simple obesity rather than leptin resistance. In obese patients, a higher level of leptin is found to cause an increase in basal ventilation associated with excess body mass, with OHS patients exhibiting an even higher serum leptin level than eucapnic individuals matched for body mass index (Phipps et al., 2002). In animal models, HFD rats exhibit an unchanged (Olea et al., 2014) or enhanced basal V_E_ (Ribeiro et al., 2017). Importantly, Ribeiro et al. found 3 weeks of HFD blunted leptin responses to hypoxia in the CB, probably due to development of CB leptin resistance, suggesting at least 3 weeks required for the establishment of leptin resistance. However, in the present study, hyperleptinaemia and leptin resistance did not occur in the LHZR most likely because of the genetic manipulation which makes the difference and the hypoventilation thus appears to be a restrictive ventilatory pattern. Another explanation may be supported by previous findings suggesting that elevation of leptin levels is a consequence of hypoxia and not of fat accumulation (Tatsumi et al., 2005). Taken together, our findings support that the impaired basal ventilation and HVR in OZRs is ascribed mainly to ob-R deficiency rather than mere obesity. We did not measure chest wall mechanics and compare the difference in chest wall impedance between two obese rats, whereas it would be expected that obesity-induced increase in chest wall impedance must play a relatively small part in such effects. This also helps better understanding of the results that ΔV_E_ induced by hypoxia is insignificant between LZRs and LHZRs.

Leptin's actions involve fast or slow onset, thus requiring minutes, several hours, even days before major changes occur. Its acute effects on breathing has been implicated in prior studies (Chang et al., 2013; Olea et al., 2015; Pye et al., 2015; Ribeiro et al., 2017). For example, Olea et al. have found that acute application of leptin in anaesthetized animals augmented basal V_E_ and potentiated the ischemic hypoxia-induced V_E_ in a dose-dependent manner (Olea et al., 2015). More recently, Ribeiro et al. reported that leptin increases V_E_ both in basal and hypoxic conditions in control rats but such effects were blunted in high fat diet fed rats (Ribeiro et al., 2017). In contrast, acute application of leptin in isolated CB type I cells failed significantly to alter the resting membrane potential and acidification-induced depolarization was unaffected by leptin, thereby suggesting that acute leptin stimulation did not alter CB's chemosensitivity (Pye et al., 2015). In addition to acute effects, chronic treatment with leptin *in vivo* also has been shown to potentiate respiratory chemoreflex (Bassi et al., 2014). Acute or chronic actions may be mediated by different leptin signaling pathways. Chronic administration of leptin in the present study did not augment basal ventilation but potentiated HVR in conscious rats, an effect persisting for ≥7 days. This appears to play an essential role in reinforcing ventilation to supply more oxygen when challenged by hypoxia. The plasma levels of leptin after 7 days treatment are quite similar to those quantified in plasma for the OZRs, taken together with the enhancement of HVR with 7 day leptin administration and the impairment of HVR in OZRs rats, indicates that 7 day stimulatory effects of leptin did not result in leptin resistance.

The reason why the basal ventilation was herein not enhanced by the chronic application of leptin would be attributable to animal's state (anesthetized vs. conscious) and the dose of leptin administered. The dose of leptin applied to our animals was chosen based on what was chronically applied previously (Wjidan et al., 2015), and lower than that used in prior reports examining the acute effects of leptin on cardiovascular (Rahmouni et al., 2002; Rahmouni and Morgan, 2007) and respiratory functions (O'Donnell et al., 2000; Bassi et al., 2012). Moreover, in the case of potentially therapeutic utilizations, this dose would be expected to specifically exert a respiratory but not excessive cardiovascular effects. Higher concentrations of leptin would be expected to saturate plasma carrier molecules and some of unbound leptin would be degradated. Furthermore, since the amount of leptin to cross blood-brain barrier is relied on receptor-mediated transport mechanism (Morris and Rui, 2009), access to the brain should be limited somehow.

Leptin's intracellular signal transduction has been extensively investigated, with exception of molecular mechanisms underlying its effect on respiratory chemosensitivity. It remains poor understood how the activation of leptin signaling affects O_2_-sensitive channels which may determine CB's chemosensitivity. Along with recent studies indicating that the chemosensitivity of CBs is closely associated with TASK-1 and TASK-3 channels (Trapp et al., 2008; Tan et al., 2010), TASK-2 channels has also been evidenced to set central respiratory CO_2_ and O_2_ sensitivity (Gestreau et al., 2010). In addition, activation of ob-Rs appears to regulate ion channels including ATP-sensitive K^+^ channels and voltage-gated K^+^ channels (Gavello et al., 2016). Although STAT3, SOCS3, and ERK1/2 may mediate leptin's role in the CBs, the critical information is lacking to data concerning modulatory effects of these molecules on TASK channels. In the present study, we did not directly address how the activation of ob-Rs and downstream signaling proteins regulated TASK-1, TASK-3, and TASK-2 channels, but notably, the altered expression levels of these channels would be expected to be attributable to leptin signaling and contribute to leptin-stimulated facilitation of the HVR. Future work is required for revealing such mechanisms.

In summary, leptin signaling participates in setting CB's O_2_ sensitivity probably through the modulation of TASK-1, TASK-3, and TASK-2 channels and thus contributes to the potentiation of HVR. This line of cellular evidence extends our understanding of molecular mechanism of leptin action on breathing, shedding light on the etiology of obesity-related hypoventilation or apnea.

## Author contributions

FY, JF, and HW acquired the data; FY, JF, and ZW analyzed and interpreted data; FY, XZ, and HY drafted the manuscript; FY, SW, and YZ were responsible for study concept and design; YZ and SW obtained research funding.

### Conflict of interest statement

The authors declare that the research was conducted in the absence of any commercial or financial relationships that could be construed as a potential conflict of interest.
