# Supplementary material for: Leptin Signaling in the Carotid Body Regulates a Hypoxic Ventilatory Response Through Altering TASK Channel Expression
Source: Front Physiol. 2018 Mar 27;9:249. doi: 10.3389/fphys.2018.00249 (PMC5881163; doi:10.3389/fphys.2018.00249)
Supplement: Supplementary file 1 [file Presentation1.PDF]

Supplementary files.

1. Original gel images showing downregulation of pSTAT3/STAT3 and TASK-1,-2, -3 channels in CBs

Ob-Rb

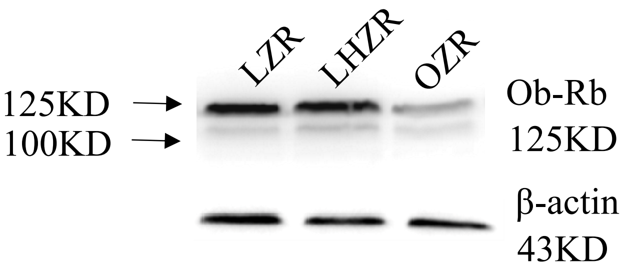

TASK-1

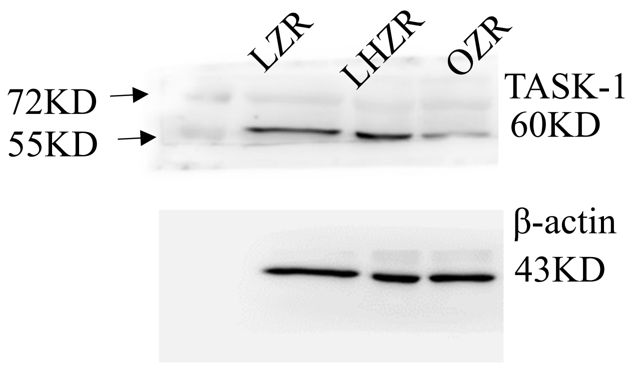

pSTAT3

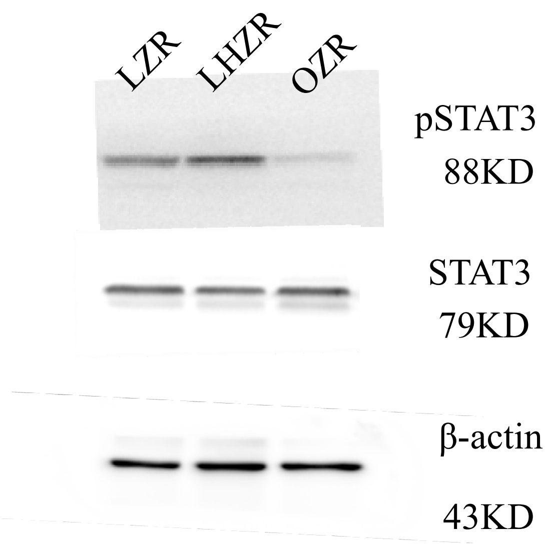

TASK -2

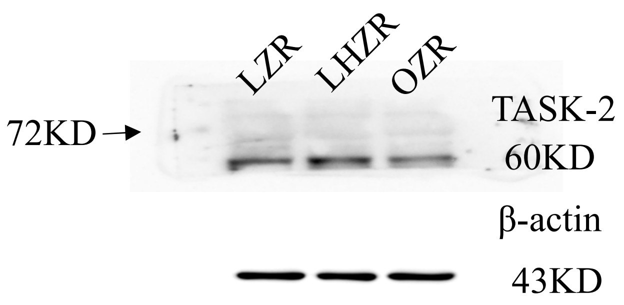

TASK-3

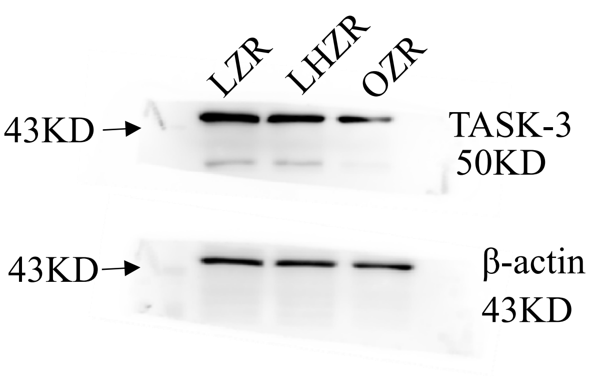

2. Original gel images showing stimulatory effect of leptin on pSTAT3/STAT3 and TASK-1, -2 -3 channel protein expression in CBs

Ob-Rb

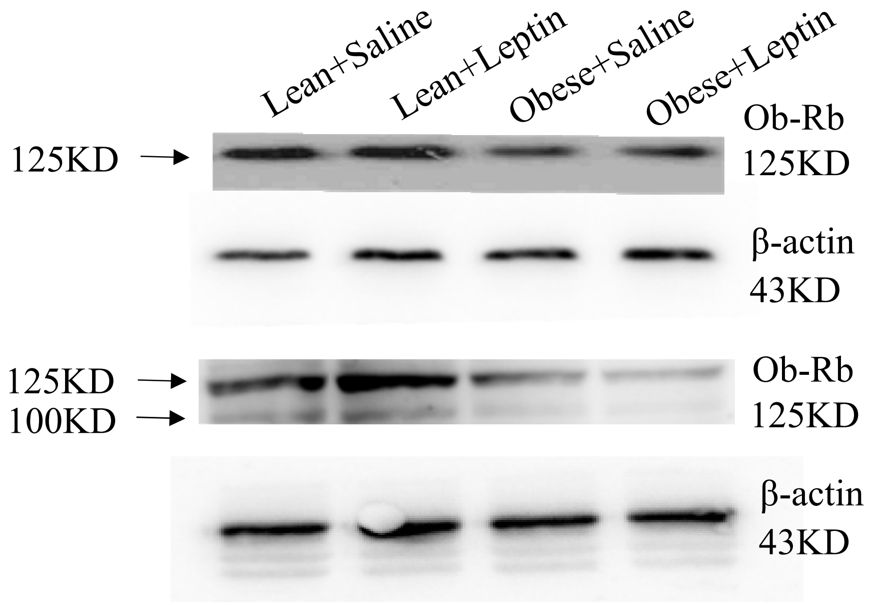

pSTAT3

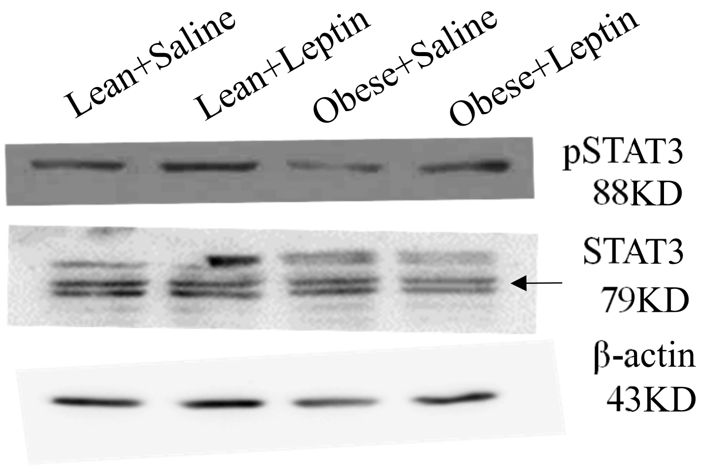

TASK-1

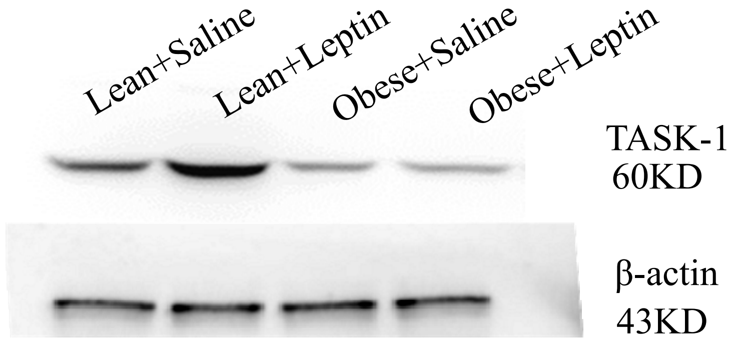

TASK -2

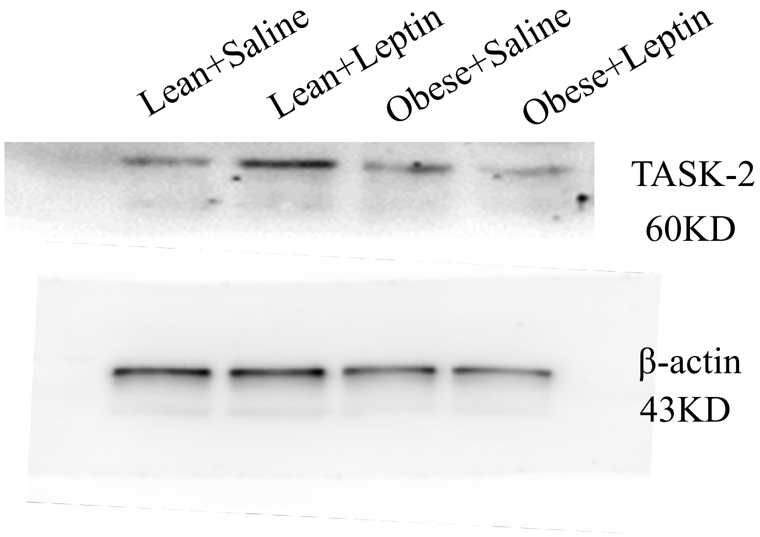

TASK-3

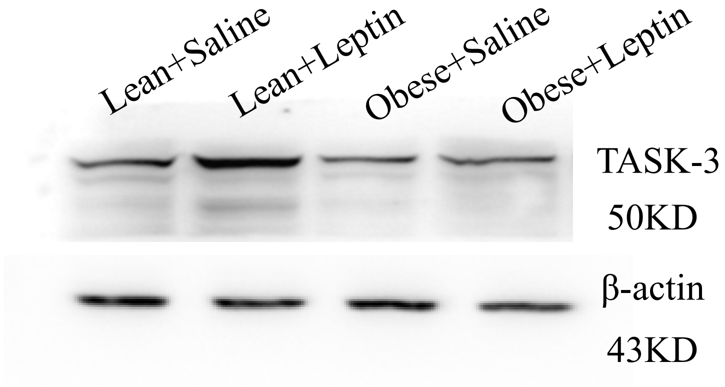

Lean+Saline, LZR with hypodermic injection of Saline;
Lean+Leptin, LZR with hypodermic injection of Leptin;
Obese+Saline, OZR withwith hypodermic injection of Saline;
Obese+Leptin, OZR withwith hypodermic injection of Leptin.
*Note: The data herein obtained in Obese+Saline and Obese+Leptin groups were not presented in the manuscript because of minor importance.
